# Assessment of Knowledge, Attitude, and Practice Regarding Topical Corticosteroid Use Among Dermatology Outpatients in a Tertiary Care Hospital: A Cross-Sectional Study

**DOI:** 10.7759/cureus.101743

**Published:** 2026-01-17

**Authors:** Samiksha Agarwal, Apurva Agrawal

**Affiliations:** 1 Pharmacology, Rabindranath Tagore Medical College and Hospital, Udaipur, IND

**Keywords:** awareness, dermatology, misuse, steroid abuse, topical corticosteroids (tcs)

## Abstract

Introduction: Topical corticosteroids (TC) play a vital role in the treatment of many diseases and have been prescribed extensively. Over-the-counter availability and self‑medication by patients make them one of the most commonly misused medications. As TC misuse is associated with serious adverse effects, it is important to know how much patients are aware of them.

Aim and objective: The aim of this study was to evaluate knowledge, attitude, and practice (KAP) related to TC use among patients attending the dermatology outpatient department of a tertiary care government institute located in southern Rajasthan, India.

Methods: A cross-sectional questionnaire-based study was conducted on patients aged 18 years and above who have been prescribed at least one TC. A total of 100 consenting patients were interviewed on the basis of a structured questionnaire containing 15 questions regarding sociodemographic profile, self-reported knowledge of TC application, use, and misuse.

Result: Of the 100 participants, 60% were male, and 50% belonged to the age group of 18-39 years. Thirty-four (34%) were illiterate, and 39% had only a school education. Dermatitis (37%) was the most common condition, and betamethasone (68%) was the most common TC prescribed. The majority self-reported that they knew the appropriate quantity (88%) to be applied, as well as the frequency (89%), and duration (84%) of application. Of the participants, 70% were unaware of the potential risks of unsupervised TC use, 47% believed that the same TC could be shared with family, 82% had previously used topical medications without consultation, 85% had stored topical medications for future use, and 70% were unaware of the dangers associated with TCs.

Conclusion: High-potency TCs are commonly prescribed. Awareness regarding the safe use of TCs is lacking in the majority of patients receiving them. Healthcare providers need to actively educate patients regarding TCs and the impact of their misuse.

## Introduction

Topical corticosteroids (TCs) are widely used in skin diseases due to their anti-inflammatory, immunosuppressive, and anti-mitogenic properties. They are prescribed in various dermatological conditions, e.g., psoriasis, atopic dermatitis, seborrheic dermatitis, intertrigo, eczema, and lichen simplex chronicus, etc. [1]. Due to their over-the-counter availability, patient-driven self-medication, and limited healthcare access, TCs are among the commonly misused drugs. They are frequently misused for conditions where they are not indicated, including fungal skin infections, acne, and as fairness creams [2-5]. Young adults purchase over-the-counter fairness creams that contain TCs in order to subjectively feel better about their appearance [6,7].

Furthermore, topical steroids are available in various irrational fixed-dose combinations (FDCs) [8], most commonly containing a potent steroid alongside one or more antifungals or antibacterials, which increase the risk of severe, irreversible cutaneous side effects [9]. Adrenal suppression, purpura, striae, steroid-induced rosacea, perioral dermatitis, hypertrichosis, and epidermal and dermal thinning are the cutaneous and systemic side effects that can arise from improper and excessive usage of TCs [10]. In a rural north Indian outpatient study, 29.2% patients were reported to misuse TCs [11].

Given the established high frequency of TCs prescription and abuse, investigating patient awareness regarding appropriate usage is critical to assessing the local risk profile and guiding educational interventions within the southern Rajasthan region. Although numerous studies on TC misuse have been reported internationally and nationally [1,10-14], data specifically concerning patients prescribed TCs in southern Rajasthan remain limited. Thus, this study was planned to assess the knowledge, attitudes, and practice (KAP) of patients regarding the use and misuse of prescribed TCs.

## Materials and methods

Study design and setting

This was a cross-sectional questionnaire-based study, conducted in the dermatology outpatient department (OPD) of Rabindranath (RNT) Medical College, Udaipur, Rajasthan, India, from November 10, 2024, to December 10, 2024. The study was approved by the Institutional Ethics Committee, RNT Medical College & Controller & Attached Hospitals (IEC/2024/415). The study was conducted in accordance with the Good Clinical Practice Guidelines and the Indian Council of Medical Research Guidelines. Written informed consent was taken from all participants.

Study population and sample size

All patients of either sex, aged 18 years and above, who were prescribed at least one TC and who provided written informed consent were included in the study as participants, using a convenience sampling strategy. Patients with psychiatric illness, inability to communicate, or incomplete questionnaire responses were excluded.

Sample size was calculated using Cochran’s formula for cross-sectional studies. The proportion stated by Mahar et al. [15], based on 72.8% of patients who were prescribed betamethasone, was used to calculate the sample size. The sample size was 84 patients, with a 10% absolute error and a 90% confidence interval, which was rounded off to 100 patients.

Data collection and study tools

A structured questionnaire consisting of 15 questions was developed in English (see Appendices) after a thorough literature search on previous studies [1,12,16,17] conducted in other regions. The questionnaire was reviewed and validated by the faculty of the Department of Pharmacology and Dermatology. It consisted of three sections: (i) informed consent, (ii) socio-demographic data, and (iii) KAP regarding use, misuse, storage, future use, and adverse effects of TCs. The patients were briefly explained about the purpose of the study in the local language, ensuring clarity and understanding. They were informed that participation was voluntary, and they could withdraw at any stage without any consequences. Each interview was conducted face-to-face in their local language by trained investigators in a private area of the OPD to ensure confidentiality and to minimize interviewer bias. On average, each interaction took around 10-15 minutes. Demographic information such as age, gender, education, occupation, and socioeconomic status was recorded for each participant to assess possible correlations with awareness level.

Statistical analysis

Descriptive statistics were used to summarize the data. Categorical variables were expressed as frequencies and percentages, and continuous variables were summarized using mean ± standard deviation (SD). To determine the association between sociodemographic factors and participant responses, the Chi-square test was employed. A p-value of less than 0.05 was considered statistically significant.

## Results

A total of 100 participants were included in the study, out of which 60 were male, and 50 were young adults (18-39 years) (Table 1).

**Table 1:** Sociodemographic profile of participants (N=100)

Parameter		Number
Sex	Female	40
	Male	60
Co-morbidities	Yes	41
	No	59
Marital status	Married	79
	Unmarried	21
Education	Illiterate	34
	Matriculate	39
	Graduate	18
	Post graduate	9
Monthly family income (Rupees)	0-10,000	22
	10,000-25,000	29
	25,000-50,000	19
	>50,000	30
Age group (years)	18-39 (Young adult)	50
	40-59 (Middle-aged)	26
	>59 (Elderly)	24

**Figure 1:**
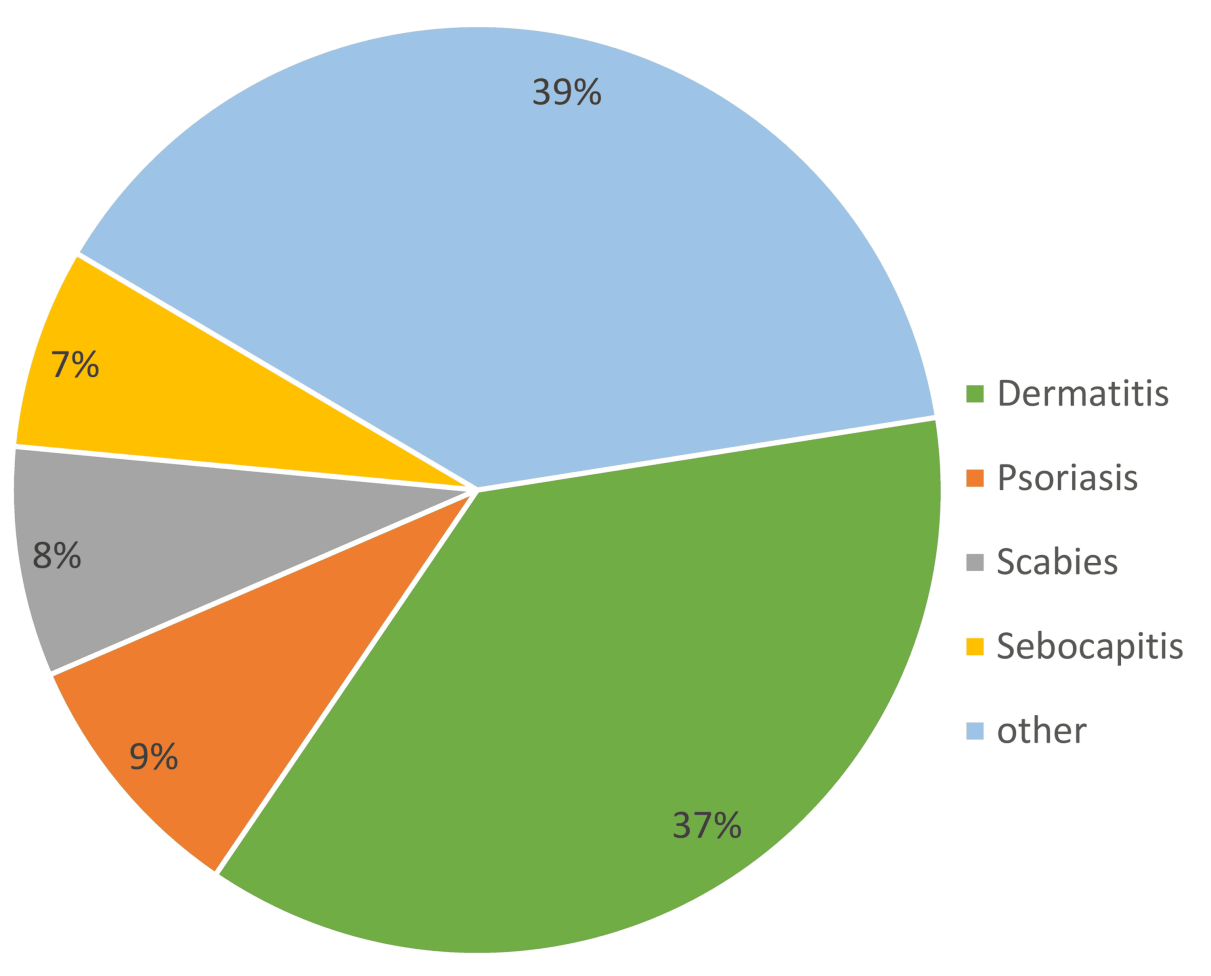
Distribution of skin conditions for which topical corticosteroids (TCs) were prescribed (N=100)

**Figure 2:**
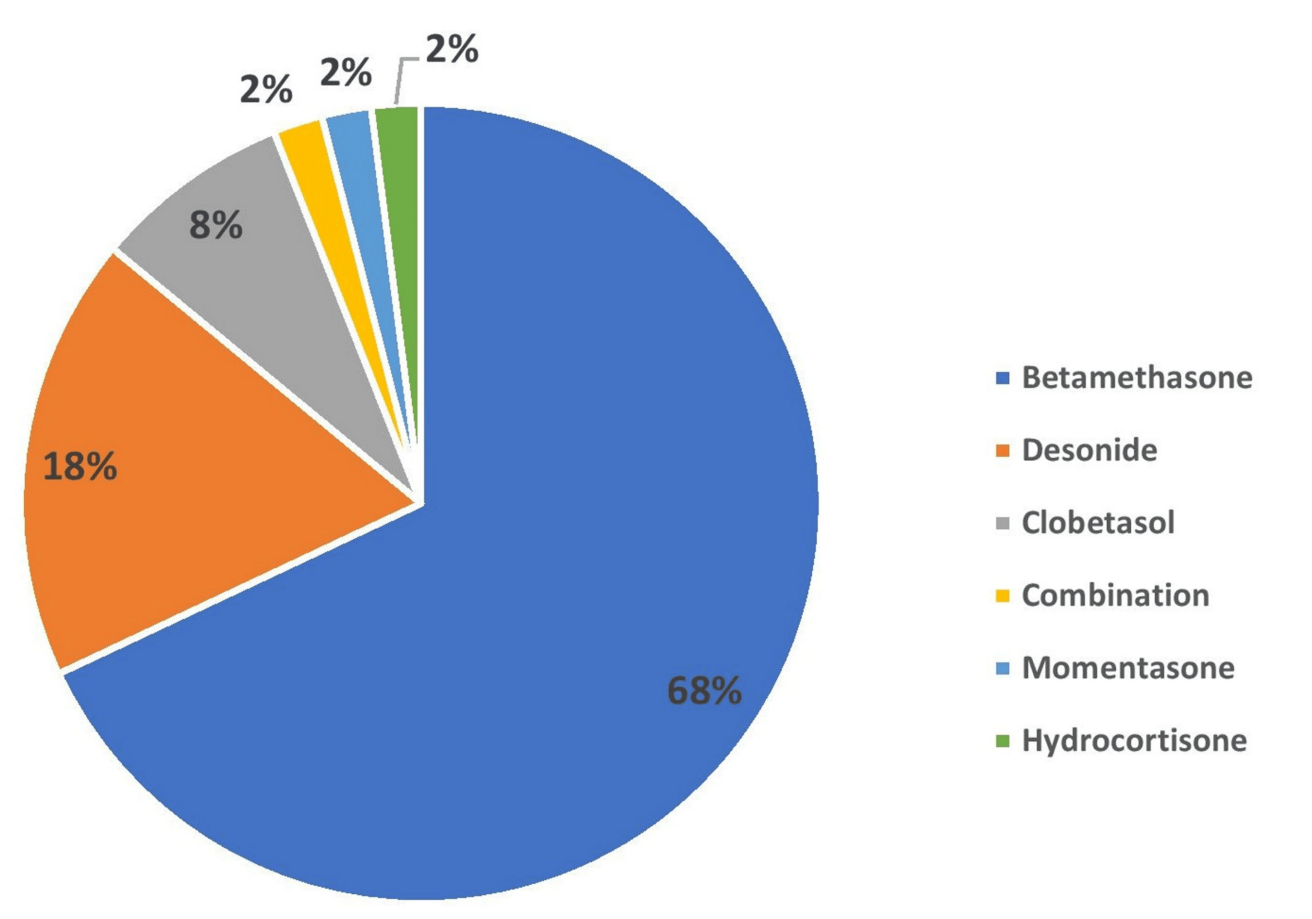
Distribution of topical corticosteroids (TCs) prescribed (N=100)

The most common skin condition for which TCs were prescribed was dermatitis (37%), followed by psoriasis (9%) (Figure 1). The most commonly prescribed TC was betamethasone (68%), followed by desonide (18%) (Figure 2).

A total of 88 participants responded that they knew the quantity of the drug that had to be applied, 89 respondents knew the suggested frequency of application, and 84 participants were aware of the proper duration for which the drug had to be applied. Of the participants, 47 responded that they would use the same steroid formulation on another family member for a similar appearing skin condition, and 82 acknowledged using topical medicinal creams, ointments, or lotions without prior consultation with a healthcare professional. A total of 70 participants had not heard the term 'steroid' and were unaware of its adverse effects (Table 2). Self-reported knowledge regarding drug application (all parameters) was statistically significantly associated with family income. Usage of topical preparations without a doctor’s consultation was more common in the low-income group, and unawareness regarding the adverse effects of steroids was statistically significantly higher in the participants with low-income, illiterate, age ≥ 40 years, and those with comorbidities (Table 2).

**Table 2:** Participant’s knowledge, practice, and attitude regarding topical corticosteroids (TCs)

Questions	Gender, n (%)			Marital status, n (%)			Literate status, n (%)			Income (INR), n (%)			Age (years), n (%)			Comorbidity, n (%)		
	Male	Female	p value	Married	Un-married	p value	Illiterate	Literate	p value	< 25000	≥ 25000	p value	< 40	≥ 40	p value	Present	Absent	p value
Knowledge of Proper Application																		
Knew the correct amount of TC to apply (n=88)	55 (91.6)	33 (82.5)	0.77	71 (89.8)	17 (80.9)	0.26	28 (82.3)	60 (90.9)	0.21	40 (78.4)	48 (97.6)	0.002	45 (90)	43 (86)	0.001	36 (87.8)	52 (88.1)	0.96
Knew the appropriate frequency of TC application (n=89)	54 (90)	35 (87.5)	0.69	72 (91.1)	17 (80.9)	0.18	28 (82.3)	61 (92.4)	0.12	41 (80.3)	48 (97.6)	0.005	45 (90)	44 (88)	0.74	37 (90.2)	52 (88.1)	0.74
Knew the recommended duration of TC use (n=84)	52 (86.6)	32 (80)	0.37	69 (87.3)	15 (71.4)	0.07	25 (73.53)	59 (89.3)	0.04	36 (70.5)	48 (97.6)	0.0001	41 (82)	43 (86)	0.58	36 (87.8)	48 (81.3)	0.38
Practices and Attitude																		
Used TC for another family member with a similar condition (n=47)	31 (51.6)	16 (40)	0.25	37 (46.84)	10 (47.6)	0.94	23 (67.6)	24 (36.3)	0.002	27 (52.9)	20 (40.8)	0.22	24 (48)	23 (46)	0.84	21 (51.2)	26 (44.07)	0.48
Used topical medications without consulting a doctor (n=82)	46 (76.6)	36 (90)	0.089	64 (81.01)	18 (85.7)	0.61	30 (88.2)	52 (78.7)	0.24	48 (94.1)	34 (69.3	0.0012	40 (80)	42 (84)	0.60	34 (82.9)	48 (81.36)	0.84
Stored leftover TC for future personal/family use (n=85)	49 (81.6)	36 (90)	0.25	68 (86.08)	17 (80.9)	0.55	31 (91.1)	54 (81.8)	0.21	46 (90.2)	39 (79.5)	0.13	42 (84)	43 (86)	0.77	36 (87.9)	49 (83.05)	0.51
Had not heard the term “steroid” or were unaware of associated adverse effects (n=70)	38 (63.3)	32 (80)	0.074	58 (73.4)	12 (57.1)	0.14	33 (97.06)	37 (56.06)	0.000023	49 (96.08)	21 (42.8)	0.00001	30 (60)	40 (80)	0.029	35 (85.3)	35 (59.3)	0.005

## Discussion

Topical steroids are an important class of drugs for the treatment of skin disorders, but they are also liable to abuse and are associated with serious adverse effects. This study provides insight into the KAP regarding TCs among patients who have been prescribed TCs. High-potency TCs were commonly prescribed. Though participants were aware (self-reported) of the application method of the prescribed TCs, knowledge regarding safe use of TCs and the harms of misuse was significantly lacking.

The most common skin condition for which TCs were prescribed was dermatitis (37%), followed by psoriasis (9%). This pattern is similar to that reported by Karekar et al. [1] and Meena et al. [5], where dermatitis and eczema were reported as the leading indications. The most commonly prescribed TCs in our study were betamethasone (68%), followed by desonide (18%). Betamethasone is a high-potency steroid and was prescribed to more than half of the participants. Use of high potency TCs has been reported by other Indian authors also [1,18]. Nerurkar et al. have reported clobetasol as the most common TC, followed by mometasone [19]. The regional variation in the choice of TCs may reflect differences in prescribing preferences and availability, but across all studies, the predominance of potent and super-potent corticosteroids indicates a worrisome trend toward stronger formulations.

The majority of the participants believe that they knew how to use and had the knowledge regarding the dose, duration, and frequency of application of TCs. The knowledge regarding the use of prescribed steroids was affected by literacy level, family income, and age. Our results suggest that educated, mature, and higher-income group patients are more vigilant about the instructions given to them regarding the use of prescribed medicines, though we were not able to confirm that their knowledge was correct or as advised by the prescriber. Proper application of TCs is critical for their effectiveness, and appropriate patient education ensures that patients apply the drug not too thinly or not too thickly over the skin [5,20]. Some studies have also reported correct application knowledge in 70-80% of participants, indicating slightly better awareness in our population, possibly due to consistent physician counselling and because they were interviewed in the outpatient department, just after they were prescribed drugs [1,21].

However, a big gap was found between the knowledge of TCs application and awareness regarding its risks and possible adverse effects. The majority of the participants (70%) had never heard the name of steroids and were unaware that steroids could be dangerous and should not be used without consulting a doctor. Karekar et al. have reported that 5.5% of participants in their study were aware that they were prescribed steroids, and less than 6% were aware that TCs can cause adverse effects [1]. Similar poor awareness levels were reported in other Indian [22-24] and international studies [12-14,25,26], where less than 10-15% of participants recognized that TCs could be harmful. These consistent findings suggest a deficiency noted globally in patient education regarding the potential risks of unsupervised steroid use.

Patients with lower literacy (p < 0.001) and socioeconomic status (p < 0.001) were largely unaware of the term ‘steroids’ and its adverse effects, and were more liable to use topical medications without consulting a doctor, highlighting the vulnerability of these populations to misuse and adverse outcomes. Patients from lower socioeconomic strata, illiterate, elderly, and those with co-morbid conditions are at higher risk of adverse drug effects due to easy availability of over-the-counter medicines [27].

In our study, 82% participants admitted that they had used topical medicines in the past without prior consultation, and 85% responded that they stored such leftover medicines for future use for themselves and family members. Patients generally do not consider topical medicinal preparations as harmful and are unaware of the risks associated with their misuse. This finding becomes more dangerous, looking to the fact that the most commonly prescribed TC in our study was betamethasone, which is a high-potency steroid. In a study by Meena et al., more than 80 different TCs containing preparations were found to be misused, the majority of them containing potent and superpotent TCs [5]. Self-medication of topical medicinal preparations is highly concerning, as nearly half of the respondents believed that it was appropriate to use the same topical medicine for other family members with similar symptoms, showing widespread acceptance of unsupervised use. The practice of using TCs for family members and self-medication without consultation was significantly higher among illiterate participants (p < 0.01), emphasizing that inadequate health literacy plays a major role in irrational TC use.

The findings of our study indicate that prescribers are giving instructions to patients regarding proper application of TCs, but there is a compelling need to also make the patients aware of the risks associated with TCs. It is important that prescribers specifically educate patients that TCs should not be stored at home and should not be used without consultation. Every consultation should be seen as a chance to educate patients, not just prescribe. Simple things like pamphlets, counselling, and clear instructions can go a long way in helping patients use TCs safely. Pharmacists can also play an important role in sensitizing patients about TCs and the dangers associated with their misuse. Targeted educational interventions focusing on illiterate and low-income populations, along with strict regulation of over-the-counter availability of potent TCs, are urgently needed to curb misuse and prevent steroid-related adverse effects.

Strengths and limitations

Our study has evaluated the self-reported awareness of patients regarding TCs prescribed to them as well as their knowledge and perception related to use, miss-use and possible risks of TCs, which has been largely unexplored in other studies.

This was a single-centre study with a relatively small sample size, which may limit the generalizability of the findings. The study relied entirely on patients’ self-reported knowledge of application practices of TCs. We were unable to independently verify these responses against actual prescriptions or observed practices, which may introduce reporting bias. Larger multicentric studies involving the general population are recommended to obtain more comprehensive and objective insights into awareness and safe use of TCs.

## Conclusions

High-potency steroids are the most commonly prescribed steroids in dermatology outpatients. Awareness regarding the safe use of TCs and potential adverse effects associated with their misuse is lacking in the majority of patients receiving steroids. Dermatologists, pharmacists, and other healthcare providers need to actively educate patients regarding safe use and avoidance of misuse of TCs.

## Appendices

Study questionnaire 

**Table 3:** Questionnaire section on demographic details of the participants.

Sr.No.	Demographic details	Response
1	Patient’s Unique Number:	
2	Age:	
3	Sex:	
4	Date:	
5	Diagnosis:	
6	Co-morbid Disease(s):	
7	Marital Status:	
8	Occupation:	
9	Education:	☐ Illiterate (IL) ☐ Matric (M) ☐ Graduate (G) ☐ Postgraduate (PG)
10	Family Income (₹ thousand/month):	☐ 0–10 ☐ 10–25 ☐ 25–50 ☐ >50

**Table 4:** Questionnaire section for the assessment of knowledge, practice, and attitude of participants.

Q. No.	Question	Response Options
1	Do you know how much quantity of the given topical drug has to be applied on the affected area?	☐ Yes ☐ No
2	Do you know how many times a day the given topical drug has to be applied?	☐ Yes ☐ No
3	Do you know how many days the topical drug has to be applied on the affected area?	☐ Yes ☐ No
4	Do you know how to apply the given topical preparation?	☐ Yes ☐ No
5	The prescribed topical drug can be applied on another family member for a similar problem?	☐ Yes ☐ No ☐ Do not know
6	What will you do with the remaining drug after the course is finished?	☐ Store it for my future use ☐ Store it for family members ☐ Return to pharmacist ☐ Throw in dustbin
7	Have you ever used topical medicinal cream/ointment/lotion without consulting a doctor?	☐ Yes ☐ No
	If Yes, name of preparation:	
8	Have you heard the term ‘steroids’?	☐ Yes ☐ No
9	Have you ever used a topical steroid without consulting a doctor?	☐ Yes ☐ No
10	Steroid can be purchased and used without consulting a doctor?	☐ Yes ☐ No ☐ Do not know
11	Topical steroids can be dangerous if used without consulting a doctor?	☐ Yes ☐ No ☐ Do not know
12	Topical steroids can cause local adverse effects?	☐ Yes ☐ No ☐ Do not know
13	Topical steroids can cause systemic adverse effects?	☐ Yes ☐ No ☐ Do not know
14	Can you name any adverse effect caused by topical steroid?	
15	Most common steroid prescribed (as per record):	
